# An in silico method for detecting overlapping functional modules from composite biological networks

**DOI:** 10.1186/1752-0509-2-93

**Published:** 2008-11-01

**Authors:** Ioannis A Maraziotis, Konstantina Dimitrakopoulou, Anastasios Bezerianos

**Affiliations:** 1 Department of Medical Physics, School of Medicine, University of Patras, GR26500 Patras, Greece

## Abstract

**Background:**

The ever-increasing flow of gene expression and protein-protein interaction (PPI) data has assisted in understanding the dynamics of the cell. The detection of functional modules is the first step in deciphering the apparent modularity of biological networks. However, most network-partitioning algorithms consider only the topological aspects and ignore the underlying functional relationships.

**Results:**

In the current study we integrate proteomics and microarray data of yeast, in the form of a weighted PPI graph. We partition the enriched PPI network with the novel DetMod algorithm and we identify 335 modules. One of the main advantages of DetMod is that it manages to capture the inter-module cross-talk by allowing a controlled degree of overlap among the detected modules. The obtained modules are densely connected in terms of protein interactions, while their members share up to a high degree similar biological process GO terms.

Moreover, known protein complexes are largely incorporated in the assessed modules. Finally, we display the prevalence of our method against modules resulting from other computational approaches.

**Conclusion:**

The successful integration of heterogeneous data and the concept of the proposed algorithm provide confident functional modules. We also proved that our approach is superior to methods restricted to PPI data only.

## Background

One of the key issues left at the hands of bioinformatics to be solved, is the deciphering of the complex organization of biological networks. In recent years, many studies have focused on determining small-scale subnetworks with distinct functional role, called functional modules [1,2]. Toward this goal assistance is provided by high throughput techniques such as yeast two hybrid system [3], protein complex identification by mass spectrometry [4,5] and microarray expression profiles [6,7] that generated large amounts of data regarding proteins and genes. However, the challenging task is to integrate these data sources in a manner that will lead to more reliable and valid functional modules.

Following this concept all state-of-the-art approaches have elaborated on protein-protein interaction (PPI) networks, where the in-between interactions reflect the direct collaboration of proteins to achieve a certain task. Nevertheless, this data is flooded with many false interactions, thus it is already established that functional modules descending solely from this data are often considered as unconfident and misleading. In literature several are the studies that concentrated on an unweighted PPI graph, despite the disadvantages posed by topology. The study of Rives and Galitski applied a hierarchical clustering algorithm based on shortest-distance as a metric to unravel the modular organization of yeast network [8]. Spirin and Mirny combined clique detection, superparamagnetic clustering (SPC) and Monte Carlo optimization (MC) to identify functional modules [9]. Recently, works like the one of Xiong and colleagues detected 'hypercliques', i.e. functional modules, in the yeast protein network via an association pattern discovery method [10].

Fewer were the attempts to enrich the PPI topology with gene expression data in the form of a weighted graph. The underlying concept is that genes with similar expression profiles are under the same transcriptional control and functionally associated [11]. Nevertheless, there are many cases where functionally related genes show dissimilar or even inverse expression profiles [12]. Despite the inherent noise embedded in this data and the fact that many of the yielded interactions are indirect, it provides significant information about genes under more perturbations in comparison to PPI data [13]. Lately studies that integrated these data with various ways prevailed in terms of functional modules over other methods that used PPI or gene expression data only [14]. Expression profiles can act as reinforcement on the PPI graph resulting to more valid and densely connected modules. The concept of integration has already been examined by works like [15], which validated that the members of permanent complexes are co-expressed, whereas the scene changes in transient complexes or in PPIs resulting from yeast two hybrid assay. Earlier studies also [16,17] examined the correlation between expression levels and protein abundance. In addition, recent studies concentrated on inferring gene function based on both data sources [18-20].

Recently, the majority of contemporary studies integrate PPI and gene expression profiles to detect biologically meaningful clusters or modules [21,14,22]. However most of the applied clustering techniques suffer from serious restraints. Studies like the one of Segal and colleagues have developed a probabilistic model where the input number of clusters was predefined and proteins were assigned to one cluster only [23]. Another deficit of these methods is that they produce discrete protein clusters, depicting roughly the real network that is characterized by inter-module crosstalk and overlap among the module members. Other shorthand is that graph clustering algorithms ignore proteins that are not topologically favored, even if these interactions are experimentally proved [9,24].

In current study we propose a method for determining functional modules based on the integration of PPI and gene expression profiles. The extraction of functional modules is performed by a novel graph clustering algorithm named DetMod (Detect Modules) that overcomes all the drawbacks mentioned above. Firstly DetMod algorithm identifies valid modules and subsequently allows the modules to merge, in cases where the merging procedure leads to better results.

One of the main characteristics of DetMod is that the extracted modules may display a controlled degree of overlap concerning their members, thus the inter-module crosstalk is preserved and a more realistic estimation of the protein network is acquired. In the literature there are other algorithmic approaches that can produce overlapping modules [24,25]. However most of them suffer from certain disadvantages like in the case of [25], where there is a loss of information, since the clustering of the graph is based on the selection of a certain number of "informative proteins" and not over the whole number of proteins as in the case of DetMod. Other applications like MCODE [24], fail to associate a large number of proteins with any functional module [26,27].

We validate the functional modules extracted from DetMod through biological and topological criteria, and by comparing our method with other PPI module detecting approaches and graph clustering algorithms [28].

We prove based on data of *Saccharomyces cerevisiae *that our method provides modules with functional and topological consistency and prevails over similar studies in literature.

## Results

We realized the concept of our approach based on yeast proteomics and microarray datasets. Firstly we integrated these datasets in the form of a weighted PPI graph. Next the enriched PPI graph was partitioned according to DetMod algorithm. The proposed algorithm performs clustering on the weighted graph structure determining functional modules with controlled overlap as described in detail at the Methods section. The 335 identified functional modules were tested concerning their connectivity density, their coverage in protein complexes and their functional enrichment in biological process GO terms. At the same time we compare, by means of these validation criteria, our modules with artificial modules and modules resulting after applying MCL algorithm (called also PPI method) to our PPI dataset [29,30].

Although, during the last years several algorithms [24-26] have been applied to the problem of determining functional modules, there are studies [27] that have shown that MCL is especially efficient, compared to others, in identifying protein complexes from PPI nets. The concept of MCL is to find clusters through iterations of expansion and inflation that promote the densely connected regions and decline the sparsely connected regions, respectively. In the end the process converges toward a partition of the graph, where the high-flow regions (clusters) are separated with limits from regions with no flow.

### Data sources

We tested the performance of our method based on data of *Saccharomyces cerevisiae. *An important issue is reliability, when dealing with PPI data from high-throughput techniques [31,32,5]. We decided to use highly reliable data descending from two studies [33,34], which assigned a confidence score to every interaction. From the first study we selected interactions of high and medium confidence and excluded the low ones. The second study assigned a likelihood ratio to every interaction and we chose interactions with likelihood ratio larger than 1, a limit also regarded as reliable by the authors. After combining these data sources, we ended up with 3250 proteins and 10750 interactions among them (without self connecting links). The final network consists of a large component of 2800 proteins and 137 smaller components with less than 5 members.

The gene expression data of the corresponding proteins is derived from a study that contains cell cycle related profiles using cdc15 synchronization over three cell cycles [35]. The expression data is available in the form of a matrix with N rows and D columns. The columns represent the 24 time points and the rows the gene profiles during the cell cycle. We selected cell cycle data because it elevates the dynamic character of genes during the phases of the cycle and appoints the periodicity of specific genes at certain phases, revealing their cell-cycle regulation.

The initial stage of our approach involved the clustering of expression profiles by SSFKCN algorithm [36]. SSFKCN uses GO information to semi-supervise the clustering of gene expression profiles, and can automatically determine the number of clusters. We enhanced the performance of SSFKCN by providing GO information for 15% of the genes, to acquire more biologically valid clusters with their members sharing the same functional annotation. The algorithm resulted in 18 clusters and their functional enrichment in biological process GO terms was checked via the SGD GO Term Finder [37]. This tool showed that all clusters display statistical over-representation of GO terms beyond what would be expected by chance, with the respective p-values smaller than e-10.

Next we weighted the PPI graph according to the procedure described in the Methods section. Lastly, DetMod algorithm was applied in the enriched large component of the graph and 335 modules were determined. In these 335 modules, 2384 proteins (85.2%) of the large component are contained. In regard to the overlap displayed between modules, we set such parameters to the algorithm so that the allowed overlap was limited to 35%. After examining all 335 modules, we observed that 181 (54.2%) modules had no common members with any other modules.

### Connectivity density

The first criterion applied to our 335 determined modules is connectivity density. This metric is the ratio of the total in-module degrees of the vertices to the total number of their connections and depicts how well connected are the members of the module. Many studies have used this topological metric, which ranges between 0 and 1, and it has already been established that functional modules should have connectivity density between 0.5 and 1 to fit this definition [14]. As the value of this metric, increases more dense structures are acquired.

Apart from just analysing our modules in terms of this metric we compared their connectivity density with artificial modules. We achieved this, through a randomization procedure, where we replaced 25% of the members of the modules with others that connect to the members of the modules but do not belong to the original ones. This randomized replacement was realized iteratively 10 times for each one of the 335 modules, and the average connectivity density was estimated.

**Figure 1:**
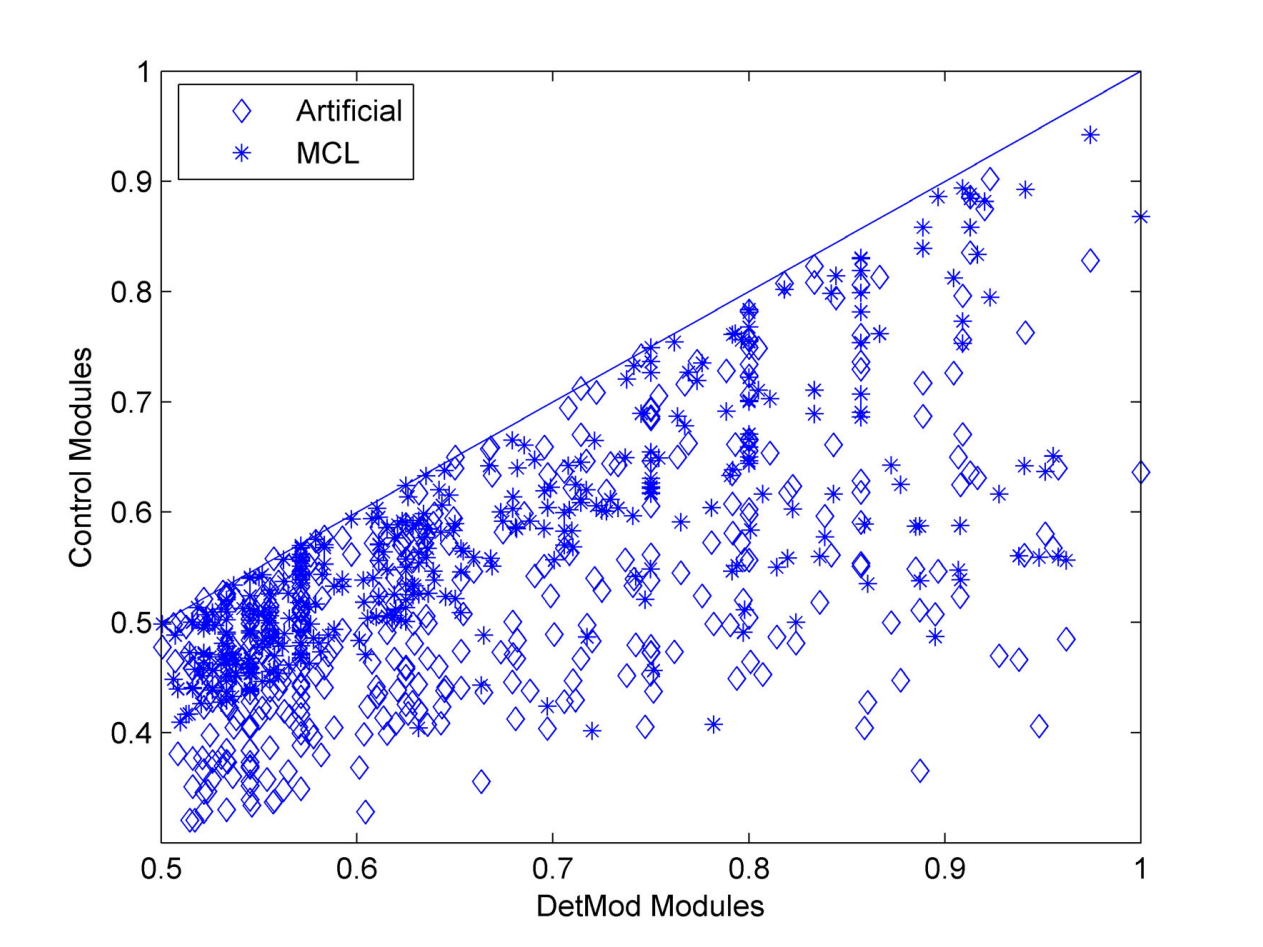
**Scatter plots of statistical metrics for DetMod and control modules (artificial and MCL).** Each data point represents statistical value for a specific DetMod functional module (x-axis) and its corresponding control modules (y-axis). The dashed line corresponds to the line y = x. When a data point is below the line then the control module has a lower statistical value than the actual one, while the opposite stands for the case a data point is above the line. When the data point is on the line it means that the derived and its corresponding control module have the same value. In the case of artificial modules each datapoint is the average of 10 randomized replacements. It is evident that all DetMod modules have better connectivity density than the control modules.

Additionally, we calculated the connectivity density of another set of modules that descended after applying the MCL algorithm to our PPI dataset. In Figure 1, we compare the density of the DetMod modules with the artificial and MCL modules (called otherwise control methods). All DetMod modules have superior connectivity density comparing to the modules of control methods. This observation elevates the ability of DetMod to produce modules with self-reliance and topological consistency.

### GO annotations

The second criterion involved the GO (Gene Ontology) annotation scheme. To gain insights into the underlying biological processes of the modules, we used the SGD GO term Finder [37]. This tool estimates the p-value of the biological process GO terms found in a module. This p-value represents the probability of observing the co-occurrence of certain proteins with the same GO annotation in a module by chance based on binomial distribution. The statistical significance of a module in a GO term is increased as the p-value gets lower.

**Figure 2:**
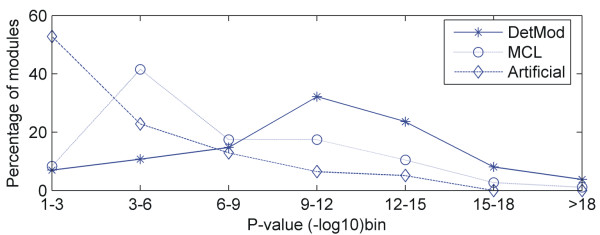
**In this diagram the functional enrichment of modules in biological process GO terms is checked by means of p-value.** As the p-value bin gets larger the more robust is a module with regard to the biological process it carries out. It is evident that the majority of control modules have p-value bins ranging between 1–12, while DetMod modules between 9 and 18.

Specifically, we examined DetMod modules and control (artificial and MCL) modules to see in which case the p-values were better. It is apparent from Figure 2, that 65% of DetMod modules have p-value bins larger than 9. On the contrary, the majority (85%) of artificial modules has p-value bins ranging between 1 and 9 and the majority (85%) of MCL modules have p-value bins fluctuating between 1 and 12. This observation is a very strong indicative that our integrated method encapsulates in the same module proteins that participate in the same biological processes and even a partial replacement of these proteins (i.e. artificial modules) ruins the functional robustness of the module. DetMod, also outperforms MCL algorithm, indicating once again that the integration of the two kinds of data and the concept of DetMod are superior to methods restricted to PPI data only.

### Protein complex overlap

**Figure 3:**
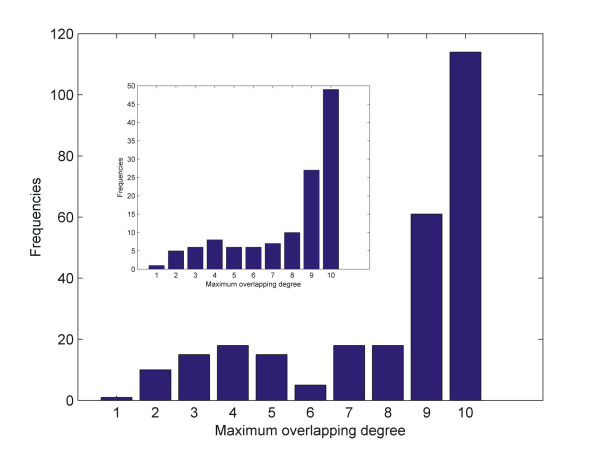
**In this histogram the degree up to which protein complexes are incorporated in DetMod functional modules is displayed.** Thus all protein complexes annotated by CYGD were set against DetMod modules and the ratio of overlap to complex was estimated. The inset histogram refers to the fraction of protein complexes with five or more protein members.

A significant validation criterion of our approach was to analyze modules in terms of protein complexes they contain. It is known that protein complexes are by definition very close to the concept of functional modules, since they represent assemblies of proteins that interact up to a great degree and carry out distinct biological activities [38]. Thus, it was important to clarify whether or not our modules encompassed whole protein complexes (annotated by the Comprehensive Yeast Genome Database at MIPS [39]) in their entirety or just parts of them. This database contains 315 protein complexes and it is worth mentioning that 188 complexes (59.7%) have less than 5 members, 127 complexes (40.3%) have 5 or more members and 111 complexes have strictly 5 members. The overlap between each complex and a functional module was identified and the degree of overlap to complex size was calculated. As it is shown in the histogram of Figure 3, 115 complexes were detected in their entirety. Knowing that small complexes with 5 or less members could be incorporated in modules by chance, we investigate separately the overlapping degree of complexes with more than 5 members (inset histogram). It is evident in this case also that 42 such complexes (33.1% of the 127 complexes) were almost completely identified while 85 complexes (67% of the 127 complexes) were found up to a great degree (80% coverage).

Additionally it was of great importance to prove that the modules of our integrated approach were biologically more meaningful than the modules extracted from other methods. In order to have a more objective comparison we have evaluated our approach and DetMod algorithm, against not only the PPI method but also with other clustering algorithms that could provide overlapping modules as well as against a method that could benefit from the enriched graph structure we have created.

A characteristic example of an algorithm that can produce overlapping clusters is the case of MCODE [24]. One major drawback of this algorithm is that a large portion of the proteins are not part of any modules. Indeed when we applied MCODE with the default settings, on our data set (results not shown) we found that more than 40% of the proteins were not members of any functional module. Next step was to compare our method against a clustering algorithm that could be applied to the enriched weighted graph we have created. An algorithm such as this is k-metis [28] that has been applied with success in various fields including the one of Systems Biology. We have applied k-metis on the weighted graph that we applied DetMod, providing this way k-metis with the same amount of information as we did for DetMod. One of the disadvantages of k-metis is that it cannot determine automatically the number of the clusters. We have run the algorithm ten different times and kept the best results.

**Figure 4:**
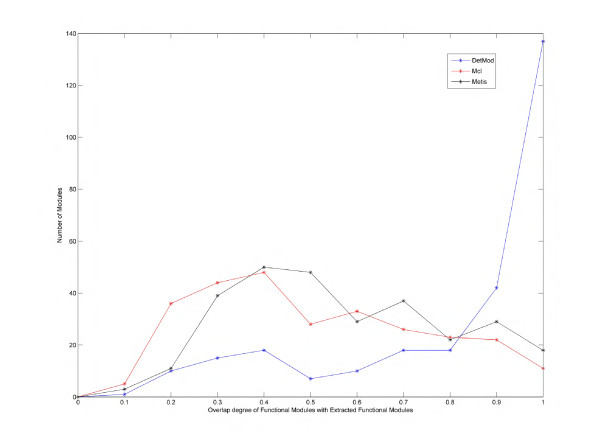
**In this plot DetMod, MCL, and Metis extracted modules are compared in terms of protein complex overlap.** In the x-axis we represent the degree of overlapping found for a specific protein complex, in a specific functional module. In the y-axis we represent the number of protein complexes found for a specific degree of overlap.

The plot of Figure 4, shows that all DetMod modules have by far higher degree of protein complex overlap than MCL modules and few are the cases were these degrees are equal. This remark corroborates that the corner-stone of our method, i.e. the integration of different kinds of data, fulfils more successfully the biological interpretation of the term 'functional module'. Additionally, DetMod algorithm proved to be able to acquire by far better results than other graph clustering algorithms, like k-metis, when given the same amount and kind of biological data.

## Discussion

In this study, we integrated gene expression and protein-protein interaction data of *Saccharomyces cerevisiae *in the form of a weighted PPI graph. Then, we applied the novel DetMod partition algorithm at the main component of the graph and 335 functional modules were identified in the yeast protein-protein interaction network. We validated these modules by examining their connectivity density, their functional enrichment in biological process GO terms and their coverage in protein complexes.

The challenging task was to unify the two types of data in a manner that would lead to more valid functional modules. Our network in particular is very complex since it consists of 3250 proteins with 10750 interactions. We chose to weight every interaction via the corresponding gene expression profiles to lessen the burden of false interactions and acquire better functional modules. Interestingly, the weight serves in many cases as a savior for interactions that are not favored topologically but are experimentally verified. After all it is already established that domain knowledge over the strength of connections can promote network analysis [40,41]. Then the DetMod clustering algorithm incorporates the weights into the partitioning process, leading to more confident modules. Following the definition of our weight metric, small weight represents enhanced relation. This means that transient or unstable interactions will have large weights due to the less correlated expression profiles. However, the proposed algorithm takes into account not only the weights but the topology as well, because it is highly desirable the obtained modules to be densely connected. Therefore, while the topology emphasizes the significance of, including highly interacting partners the weights reassure that the resulting modules will be biologically valid. Several studies like [14] have already shown that the subnetworks obtained from a weighted graph have higher probability to represent the real functional modules.

The fast DetMod algorithm we propose was proved successful in the detection of functional modules. Its concept surpasses many drawbacks of current graph clustering algorithms, such as the input number of clusters [23]. It is already mentioned that DetMod incorporates in its procedure another algorithm called DMSP, which starts from a randomly selected 'seed' protein. DetMod produces through the merging procedure, modules that display overlap concerning their members and this attribute makes our modules more realistic. In order for us to show the superiority of our integrated approach and the concept of DetMod, we applied the MCL algorithm to our PPI dataset. Furthermore, we created artificial modules, resulting from partial replacement of the members of DetMod modules. These two control methods were checked by all validation criteria as a measure of comparison.

Specifically, the first validation criterion is connectivity density, a topological metric that shows how densely connected are the modules. We proved that DetMod modules have by far improved connectivity density from both control methods. An example is the module (12 members) characterized by the GO terms mRNA processing (GO: 0006397) and mRNA polyadenylation (GO: 0006378) with p-value smaller than e-15. The second term refers to the enzymatic addition of a sequence of 40–200 adenylyl residues at the 3' end of a eukaryotic mRNA primary transcript [42]. This module has connectivity density over 0.8 and is a representative example of our approach, because it depicts the ability of DetMod to detect modules where all members share the same functional annotation. In case of another clustering algorithm where the input number of clusters is predefined, this small module would probably be part of a larger one; thus it would lose the distinct identity that has in our partitioning process. It was considered significant from the beginning that the obtained modules should contain as many members so that the connectivity density does not drop over 0.5. The identification of this module by DetMod can be interpreted byway; it incorporates members that entered the module after complying with the gene expression requirements but also its members have strong functional correlation on the PPI network.

The second criterion involves the characterization of functional modules in terms of biological process GO terms. It is essential for the modules to comprise proteins that carry out a certain task and this estimation is quantified using p-value. An example is a 12 member module characterized by the terms 'mitochondrial translocation' (GO: 0006628) and 'organelle organisation and biogenesis' (GO: 0006996) with p-value smaller than e-15 in both cases. The first term refers to the translocation of proteins across the mitochondrial membrane. In the presence of a translocating chain, the outer membrane import machinery (MOM complex) and the inner membrane import machinery (MIM complex) form translocation contact sites as a part of the membrane preprotein import machinery [43]. However this term is considered obsolete and its updated annotation is 'protein targeting to mitochondrion' (GO: 0006626) or its children. The second term is a process that is carried out at the cellular level resulting in the formation, arrangement of constituent parts, or disassembly of any organelle within a cell. It is obvious that the two terms are correlated with the second having a broader meaning.

Another interesting example is two modules (16 and 23 members) that have 3 common members and both are characterized with the GO term 'ubiquitin-dependent protein catabolic process' (GO: 0006511). It involves all the chemical reactions and pathways responsible for the breakdown of a protein or peptide by hydrolysis of its peptide bonds, starting from the covalent attachment of an ubiquitin moiety, or multiple ubiquitin moieties to the protein. One would expect that these two modules should be merged into one module but this split occurred for various reasons. Firstly the PPI data used in our study is adequate but in some cases can be proved insufficient. The two modules are topologically separated due to sparse connections between them. Additionally, these few connections had quite large weight, thus during the constructing procedure they were considered as barrier between two densely connected modules. Nevertheless, this aspect should not be regarded as a defect of our method, because the introduced merging of modules bridges the gap mentioned above. Figure 2 constitutes the proof that our modules surpass the control modules in regard to GO terms. It was expected the MCL modules would have smaller p-value bins, because this algorithm produces necessarily modules based solely on the topology. Another explicit property of DetMod algorithm is that the partitioning procedure starts from a seed protein, directing in this way the clustering process toward specific domains of the network.

Finally, we substantiated our functional modules by quantifying the overlap of our modules to well established protein complexes. There are several studies that exploit the unique nature of protein complexes to validate functional modules [44,45,14]. After all, complexes themselves can be characterized as functional modules since the two definitions are similar. An example is the 19 member module dominated by the coat complexes (COP), in which DetMod managed to encapsulate 19 out of its 25 members. The role of COPII coat (11 members) is to sprout vesicles from the ER for anterograde transport, whereas COPI coat (8 members) is responsible for retrospective transport of recycled proteins from Golgi and pre-Golgi compartments back to the ER [46]. The rest 6 proteins belong to other coat complexes that are not included to our PPI dataset. The given seed protein in this case was *SEC31 *(YDL195w) and it is worth mentioning that DetMod provided a module restricted to this complexes only, leaving no space for other proteins. Responsible for this is partially DMSP algorithm embedded in DetMod algorithm. This algorithm constructs modules around a seed protein and it is noteworthy that the size of module remained the same even after the DMSP stage. DetMod algorithm builds biologically concise modules, in contrast to other well-known graph clustering algorithms [26,47].

Another representative example is the module (7 members) characterized by the eIF3 (7 members) complex, which is responsible among with other eIFs for the initiation of protein synthesis in eukaryotic cells by stimulating the binding of mRNA and methionyl-initiator tRNA (tRNAi-Met) to 40S ribosomes to form the 48S pre-initiation complex [48]. Once again this example highlights the adaptability of DetMod against a large pool of PPIs and weights; it is of great importance for an algorithm to recognize biological frontiers encrypted both in topology and in the form of weights. Moreover, the results suggest that our modules have better complex coverage in comparison to control methods, indicating once again the capability of DetMod for detecting subnetworks that represent real functional modules. Also the prevalence of DetMod modules over the MCL modules corroborates the important role that expression profiles played for acquiring these results. This study proves indisputably the benefits gained after integrating different types of data.

Lastly an issue worth discussing is the overlap displayed between modules. Most of the graph partitioning algorithms neglect the inter-module crosstalk, which is crucial for the stability of the whole network, and provide completely separated clusters. In the path of extracting functional modules from a network, one should bear in mind that the identification and preservation of links is of equal importance. Toward this goal DetMod succeeded in detecting modules with varying overlap in terms of proteins and interactions. Besides it is already widely accepted that proteins can by nature take part in many distinct tasks, or to be members of more than one functional module. Thus, we chose to embody this network property into our integrated approach and our modules as provided are consistent with this concept.

## Conclusion

The post genomic era demands the consolidation of different types of data, which all depict the dynamics of the living cell through different perspective. This study is a proposal toward the identification of functional modules in biological networks. We successfully integrated proteomics and microarray datasets, with the first having the leading role and the second acting as reinforcement.

We have proved based on data of the model organism *Saccharomyces cerevisiae *that the novel algorithm DetMod detected highly confident functional modules onto the PPI network. Then, we examined these modules by validation criteria, which in turn substantiate that our subnetworks deserve the characterization 'functional module'. Specifically, we checked them by measuring their connectivity density, their enrichment in biological process GO terms and their coverage in protein complexes. These parameters were also estimated in two control methods, i.e. artificial modules and modules descending from a method restricted to PPI data. We observed that the obtained by DetMod modules surpass the control methods in all criteria and the difference is not random.

## Methods

At this section we analyze in detail the basic concepts of our method. Firstly we elucidate the integration of PPI and gene expression data and the reasons why this procedure can lead to biologically more meaningful functional modules. Then we describe in detail the graph clustering algorithm DetMod, which is responsible for the determination of functional modules on the PPI network. The proposed algorithm identifies functional modules on a PPI graph, which is weighted with the gene expression information. The first step of DetMod algorithm involves the construction of modules starting from a 'seed' protein. Next DetMod algorithm merges modules by examining a score we compute for each one of the extracted modules. The procedure of merging is preferred in cases, where the score of the merged cluster is better than the score of the forerunning clusters. However if the merged cluster does not significantly overlap in respect to its members with one of the forerunning clusters, then both the merged as well as the old cluster are preserved.

### Data Integration

In our work we chose to unify the above types of data for various reasons. Firstly PPI data descending from high-throughput techniques suffers from many false interactions [49]. Also protein interaction measurements stem from a certain range of experimental conditions, thus they manage to identify only a small portion of all possible protein-protein interactions. It is evident that the direction of just clustering the PPI graph (without considering gene expression data) leads to partially valid functional modules due to the exclusion of interactions that would lead to even more coherent modules. Moreover it is common among graph clustering algorithms to neglect peripheral proteins that link loosely to clusters, even if these few interactions are true and experimentally confirmed [9,24]. However an important aspect of PPI networks is that they provide information about direct partners, property lost when dealing with co-expression networks. On the other side gene expression data provides information of the genome under many different experimental conditions despite the embedded noise [13]. Although co-expression between two gene profiles implies that they are under the same transcriptional control and functionally correlated, the resulting interactions are often indirect.

Specifically we used highly confident PPI data in the form of a graph G (V, E), where vertices represent proteins and edges represent interactions. Then we applied a clustering algorithm at the respective gene expression profiles. The number of clusters was appointed both by the algorithm itself as well as by the functional enrichment of clusters in GO (Gene Ontology) terms. Next we weighted the interaction between two proteins according to the weight function:

*W *(*x*,*y*) = *n*_1 _(||*x *- *K*_*x*_||^2 ^+ ||*y *- *K*_*y*_||^2 ^)+ *n*_2_||*K*_*x *_- *K*_*y*_||^2^

||·|| stands for the distance metric and there are many metrics for measuring it, in this study we have used Euclidean distance. K_x _and K_y _symbolize the centroids of the clusters that genes x and y respectively, belongs to. The constants n_1 _and n_2 _add an extra confidence score to the factors of the weight function. They can have the same or different values according to which member (if any) of the function we want to enhance. We chose n_2 _> n_1 _(specifically n_2 _= 0.7, n_1 _= 0.3) because we consider the distance between centroids more significant comparing to the distance of each gene from its centroid. This selection was motivated by the noise (outliers) of gene expression profiles. Based on several runs of the algorithm and the corresponding results, in the current study we have set the values of the two variables as n_2 _= 0.7, n_1 _= 0.3, but in general we systematically found better results when the value of n_2 _was larger than n_1_.

The outcome of our integration method is a weighted PPI graph, at which the proposed algorithm will be applied in order to detect functional modules that are supported by both types of data.

### Basic notations

As we have already mentioned, in the approach we have followed, we combine gene expression profiles and PPI data, in the form of a weighted graph, G(V, E). By N(x) we denote the neighbours of a node x, or in other words the set of nodes that are connected to x. Then, the degree of x is equivalent to the number of neighbours of x |N(x)|. For a given subgraph G_1 _of a larger graph G we define the internal degree |N_G1_^INT^| as the number of edges connecting x with other vertices belonging to G_1 _and external degree as the number of nodes with which x is connected and exist in G but do not belong to G_1_.

The above concepts can be expanded to the weighted graphs easily. Weighted degree of a node is the sum of weights of the edges between x and its neighbours divided by |N(x)|. Weighted internal degree of a node x is the sum of weights of the edges between x and its neighbours within G_1 _over |N_G1_^INT^|:

(1) $$\beta_{G_{1}}^{INT}\left(x\right)=\frac{1}{\left|N_{G_{1}}^{INT}\right|}\sum_{y\in N_{G_{1}}^{INT}}w_{xy}$$

Correspondingly we define the term of weighted external degree.

The density of a graph G(V, E) is generally measured by the proportion of the number of edges in the graph to the number of all possible edges, which is equal to |V|(|V|-1) for an undirected graph. Weighted density of a graph or subgraph D_w_(G), is the sum of the weights of actual edges over the number of possible edges among all nodes in G:

$$D_{w}\left(G\right)=\frac{\sum_{\langle x,y\rangle \in E}w_{xy}}{\left|V\right|\left(\left|V\right|-1\right)}$$

### Detect module from 'seed' protein

DetMod incorporates in its first phase the application of another algorithm called Detect Module from Seed Protein (DMSP) [22] which operates in two phases. Firstly accepts one 'seed' protein and selects a subset of its most promising neighbours, subsequently expands this initial kernel to accept more proteins. This expansion is based on certain assumptions, concerning the number of neighbours for the specific protein as well as the weights of these connections.

DMSP algorithm initiates its function by selecting only a certain number of the neighbours of the 'seed' protein (named hereafter s). These adjacent nodes are sorted in descending degree of significance and this subset of nodes – proteins is named kernel.

The two criteria by which the original kernel is selected are the density of the kernel and the weighted internal and external degrees of it. Initially, the kernel K_s _is equal to all the neighbours of s. Then for each one of the neighbours u_i _belonging to Kernel(s) we find the N^INT^(K_s_), N^EXT^(K_s_), as well as the β^INT ^and β^EXT^. The objective for selecting the kernel of the seed node is two-fold. Firstly we check so that the number of edges of a kernel node within the rest of the kernel is larger or at least equal to the number of the edges that a node has outside the group. We accomplish this by requesting for the internal and external degrees of each node:

(2) $$IO\left(K_{s},u_{i}\right)=\frac{\left|N_{K_{s}}^{INT}\left(u_{i}\right)\right|}{\left|N_{K_{s}}^{EXT}\left(u_{i}\right)\right|+\left|N_{K_{s}}^{INT}\left(u_{i}\right)\right|}>p_{1}$$

In this study we selected p_1 _to have value over 45%. At the same time and after we have confirmed that a selected node fulfils the first condition, we request that the same node has smaller weighted internal degree than its corresponding weighted external degree. Nodes that fail to pass the above criteria are discarded, while those that do, are sorted based on the level that each one of them manages to do so.

This original subset of proteins is further distilled, in order to acquire an even more coherent kernel. This can be achieved by minimizing D_w_(K_s_) as:

(3) $$D_{w}^{\min}(K_{s})=\min(\underset{K_{s}}{\arg})D_{w}\left(K_{s}\right)$$

In this step, DMSP removes one at a time, each one of the sorted per significance nodes starting from the most insignificant until it reaches a minimum value of weighted density.

After the creation of the initial kernel, DMSP iteratively adds adjacent nodes based again on certain criteria. The depth of the neighbours (referring to the initial kernel) checked by DMSP vary per specific problem and data set, meaning that as long as the criteria we will mention are true the algorithm can go beyond the 2^nd ^and 3^rd ^level or not. The first criterion the algorithm checks is the same as the first one of the initial stage of DMSP described by (2). After this criterion has been checked then we select a node to be added to the module, if it satisfies the following:

(4) $$W_{vu_{i}}\leq p_{2}\cdot \beta_{G}^{INT}\left(v\right)$$

G is the final module that is built from the initial kernel (i.e. initially G = K_s_), we select the constant p_2 _to be anywhere between 0.9 and 1.0 (in the specific study we have set the value of p_2 _to 0.9). Ideally the value of p_3 _should be equal to 1.0 but given that we work on a real and very complex biological problem we allow the value to range down to 0.9. The experiments we have conducted showed that a lower value could create artifacts in the final determination of modules. Relation (4) states that in order for an adjacent node u_i _of some kernel node v, to become member of the module, its weight must be less or equal to a specific percentage of the weighted internal degree of node v.

At this point we should emphasize, that DMSP, uses two values describing the relation of internal and external neighbours (2) (we are referring to the value of p_1_). The distinction of this value depends on whether the current node is a direct neighbour of the kernel or not. In this way we have a two-layer scheme where we retain a looser criterion for immediate neighbours and a stricter one for the remote neighbours of the initial kernel (specifically we have set the value of p_1 _in equation (2) to 0.75 when DMSP checks for members in the remote neighbours of the initial kernel).

### DetMod analysis

In the first phase, DetMod iteratively applies DMSP to every node of the overall graph, therefore each node is regarded as a seed protein and based on this a possible functional module is created. Each newly constructed module is checked in terms of overlap with the rest of the modules that have been previously created. If this overlapping degree is above a certain threshold then the module is discarded. We give below the pseudocode for the first part of DetMod:

Procedure **Create_Basic_Modules**

1) G' = G;

2) Modules_List = Empty

3) While G' != empty

I) Retrieve randomly a node v from G'

II) Apply DMSP to create a new functional module M with v as seed, M = DMSP(G, v)

III) For all modules in Modules_List

i) Check if there is a module with more than p% overlap with M

ii) If there is, find = true then break

IV) End_for

4) If find! = true

I) Keep M in Modules_List

II) Keep v in Nodes_List

5) End_If

6) Delete v from G'

7) End while

As we have seen DetMod allows every node to be part of more than one module. In this way DetMod manages to compromise between the complexity of genes or their products and their tendency to participate in different groups towards achieving different goals. For this reason we insert the term of node score (easily extended to module score). This metric has dual purpose, it checks if the majority of the immediate neighbors of a respective node are in the same cluster as well as the repetitive appearance of a node and its immediate neighbors in many different clusters.

Node score is an expansion of the node degree term, and is related with the connectivity of a node in regard to its neighbors in every module.

**Figure 5:**
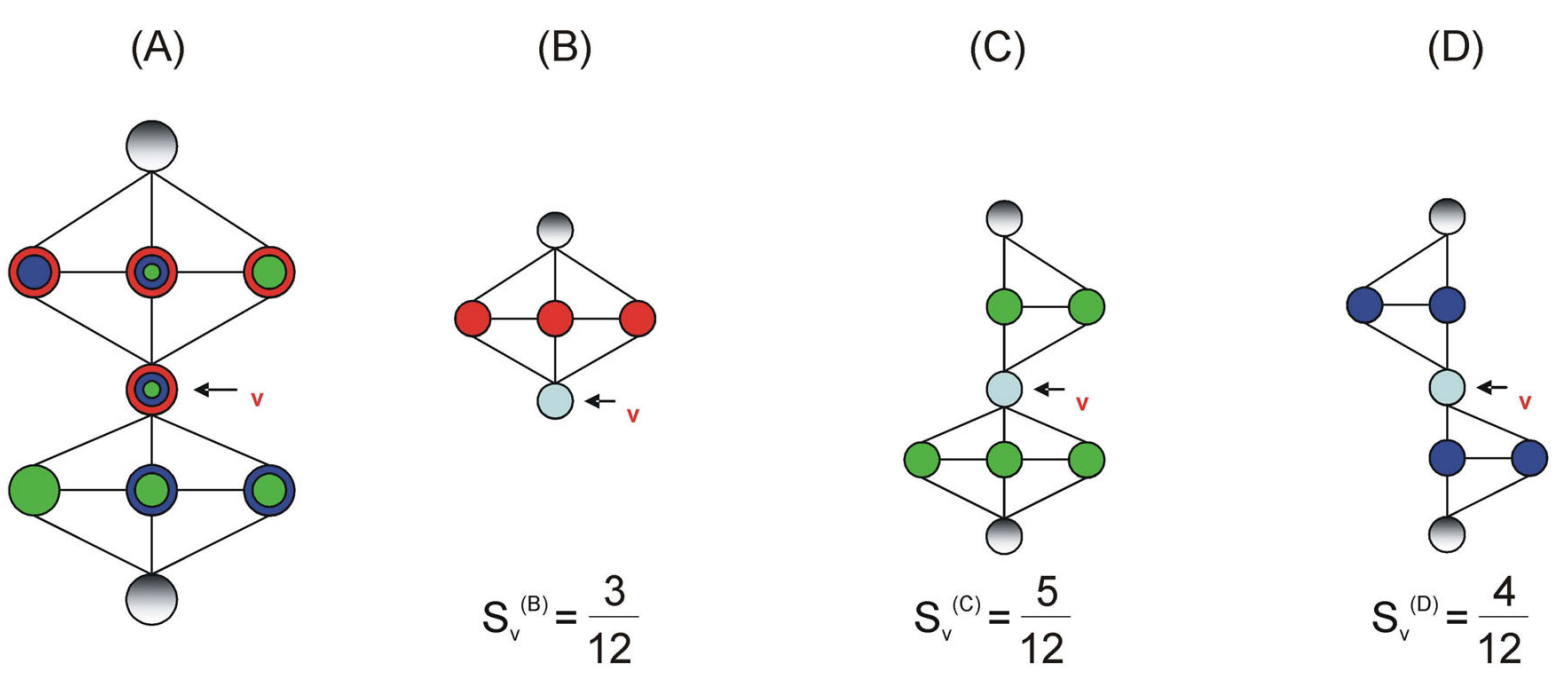
**In this figure we describe an example of the score we set to a certain node v.** In (A) we can depict the real neighborhood of v. The sub-figures in the left (B-D) are three possible modules that could be extracted from the main graph in (A). According to the neighbors of v present in each one of the modules we set a score for v in the specific module. The scores are normalized so as to have sum equal to unity. It is clear that the best score for node v is for sub-figure (C), since in this module v is connected to the majority of its neighbors.

To compute the score of a node (example given in Figure 5), we isolate the modules in which a certain node belongs, and then we check the common modules for each one of its neighbors. We add an imaginary neighbor to the total number of neighbors of v, every time the actual neighbor and the node have a common module. In mathematical terms it is:

(5) $$N_{v}^{TOT}=\sum_{i=1}^{N}\Xi \left(v,u_{i}\right)$$

with:

(6) $$\Xi \left(v,u\right)=\{\begin{array}{ll} \left|v_{C}\cap u_{C}\right|, & v_{C}\cap u_{C}\neq \emptyset \\ 1, & else \end{array}$$

where, v_c _is the set of modules the node belongs to, and N is the set of the real neighbors of v. Given the total number of neighbours for a node under the scheme we described, we can calculate the score of a node which is given as:

(7) $$S_{v}^{G}=\frac{N_{G}^{INT}(v)}{N_{v}^{TOT}}$$

After determining the score of every node we can calculate the score of a module by averaging the score of the nodes that constitute it.

In the second phase of the algorithm, the retrieved functional modules of the first phase are checked in order to determine whether or not they could be merged.

Specifically DetMod, checks every pair of connected modules, in order to determine whether or not a probable merging operation among them will lead to a new module which will have a higher score than one or both of its predecessors.

Procedure **Merge**

N = number of modules

1) For i = 1:N-1

2) For j = i+1:N

I) If Mod_i _and Mod_j _are connected

i) Mod_new _= merge(Mod_i_, Mod_j_)

ii) If Score(Mod_new_) > Score(Mod_i_) AND Score(Mod_j_)

(a) If Mod_new _has no overlap conflict save in Merge_Modules

(b) Delete the other two modules

iii) If Score(Mod_new_) > Score(Mod_i_) OR Score(Mod_j_)

(a) If Mod_new _has no overlap conflict save in Merge_Modules

(b) Delete the module with the worst score

II) End_If

3) End_For

4) End_For

## Authors' contributions

IAM designed, implemented the DetMod algorithm and the data integration method, and prepared the data sets. KD was responsible for writing the main body of the text and especially for the biological aspects of the study. All authors contributed in the preparation of the manuscript and approved its final form.
